# Obesity *versus* osteoarthritis: beyond the mechanical overload

**DOI:** 10.1590/S1679-45082014RB2912

**Published:** 2014

**Authors:** Angélica Rossi Sartori-Cintra, Priscila Aikawa, Dennys Esper Correa Cintra

**Affiliations:** 1 Faculdade Anhanguera de Campinas, Campinas, SP, Brazil;; Faculdade Anhanguera de Indaiatuba, Indaiatuba, SP, Brazil.; 2 Faculdade Anhanguera de Rio Grande, Rio Grande, RS, Brazil.; 3 Faculdade de Ciências Aplicadas, Universidade Estadual de Campinas, Limeira, SP, Brazil.

**Keywords:** Osteoarthritis, Obesity/complications, Inflammation, Leptin, Articular cartilage

## Abstract

Obesity is currently considered a major public health problem in the world, already reaching epidemic characteristics, according to the World Health Organization. Excess weight is the major risk factor associated with various diseases, such as type 2 *diabetes mellitus*, hypertension, dyslipidemia and osteometabolic diseases, including osteoporosis and osteoarthritis. Osteoarthritis is the most prevalent rheumatic disease and the leading cause of physical disability and reduced quality of life of the population over 65 years. It mainly involves the joints that bear weight - knees and hips. However, along with the cases of obesity, its prevalence is increasing, and even in other joints, such as hands. Thus, it is assumed that the influence of obesity on the development of OA is beyond mechanical overload. The purpose of this review was to correlate the possible mechanisms underlying the genesis and development of these two diseases. Increased fat mass is directly proportional to excessive consumption of saturated fatty acids, responsible for systemic low-grade inflammation condition and insulin and leptin resistance. At high levels, leptin assumes inflammatory characteristics and acts in the articular cartilage, triggering the inflammatory process and changing homeostasis this tissue with consequent degeneration. We conclude that obesity is a risk factor for osteoarthritis and that physical activity and changes in diet composition can reverse the inflammatory and leptin resistance, reducing progression or preventing the onset of osteoarthritis.

## INTRODUCTION

According to the World Health Organization (WHO), obesity has already reached epidemic proportions, affecting over one billion adults worldwide. It is the major risk factor associated with various diseases, such as insulin resistance and type 2 *diabetes mellitus* (DM2), hypertension, dyslipidemias and some kinds of cancer. Secondarily, obesity is strongly associated with a series of disturbances, like asthma and nonalcoholic steatohepatitis, in addition to osteometabolic diseases, such as osteoporosis and osteoarthritis.^(1)^ Osteoarthritis (OA) is the most prevalent osteometabolic rheumatic disease, and the main cause of physical incapacity and reduced quality of life of the population aged over 65 years. It is characterized by articular cartilage degradation.^(2)^

Historically, the relation between obesity and the development of OA was restricted to biomechanical changes in joints, caused by increased body weight, leading to the genesis of an inflammatory process in cartilages, and eventually in the development and progression of the condition. These changes occur preferably in joints that bear body weight, such as knees and hip.^(3)^ However, along with the increased prevalence of obesity in the world population, there has also been an increase in cases of OA in non-weight bearing joints, such as hand and temporomandibular (TMJ) joints.^(4)^ In this way, the influence of obesity in the development of OA is thought to go beyond joint overload due to the increase in body mass index (BMI).^(5)^

Therefore, many studies have been conducted to reveal the mechanisms involved in the relation between obesity and OA. The objective of this review was to group the most recent evidence on the possible molecular mechanisms correlated to both conditions.

### Pathophysiology of obesity and of osteoarthritis

In obesity, fat hypertrophy increases the expression and release of the so-called adipocytokines, such as interleukin (IL) 1β, IL-6, resistin, leptin and tumor necrosis factor alpha (TNF-α), that are necessary for cell differentiation and hematopoiesis, among other functions. However, such functions depend on their concentration in the bloodstream. The increase to a “super optimal” grade is already capable of beginning the low-grade inflammatory process.^(6, 7)^

These proteins reach the bloodstream and then the hypothalamus in the central nervous system, interfering in the fine regulation it exerts, by controlling hunger and energy expenditure. This disturbance makes it mainly resistant to peripheral hormones insulin and leptin.^(8)^ In this way, an individual will have uncontrolled satiety, increasing food intake, and consequently increase in weight and in fat, which, in turn, releases more leptin, reaching the level of hyperleptinemia. In high concentrations, leptin becomes inflammatory. The leptin structure is homologous to the receptors of inflammatory cytokines (IL-1 and IL-6), and therefore is considered, as a member of the superfamily of cytokines.^(7,9)^

**Figure 1:**
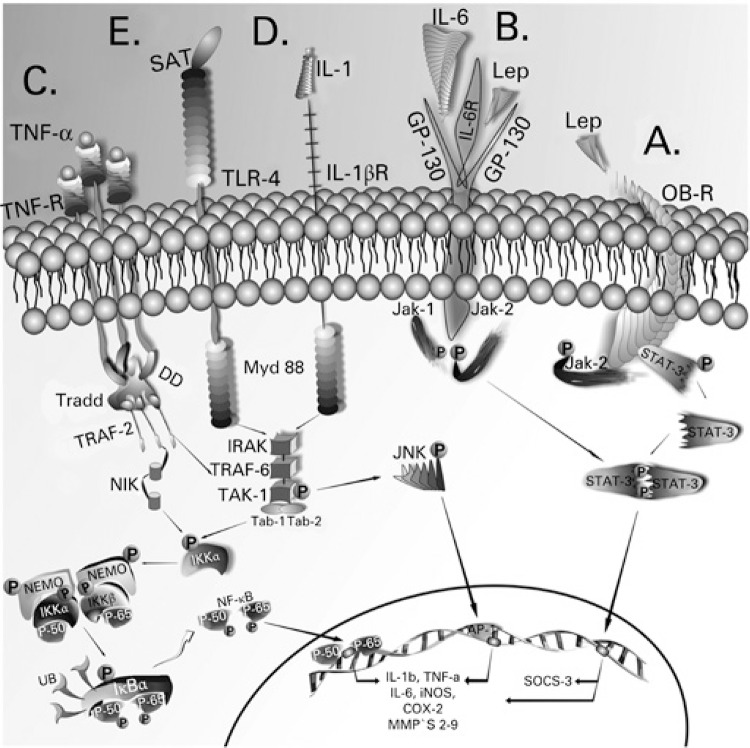
Leptin, through its receptor ObR (A) and the interleukin 6 pathway, by means of its receptor GP-130 (B), activates the transcription factor STAT3, which, in the nucleus, transcribes the gene of SOCS3 that suppresses the leptin signaling pathways. However, it also activates the transcription of metalloproteinases and aggrecanases, cartilage degradation proteins. Signaling pathways of inflammatory cytokines: tumor necrosis factor alpha through the tumor necrosis factor receptor (C), interleukin 1β by its interleukin receptor 1β (D), and the saturated fatty acids, that are recognized by the Toll-like 4 receptors (E). These pathways culminate in activation of the transcription factors NF-kβ and AP-1, that transcribe the genes of inflammatory proteins (interleukin 1β, interleukin 6, tumor necrosis factor alpha, induced nitric oxide synthase among others)

Leptin binds to its receptor (Ob-Rb), which is associated with the Janus Kinase-2 molecule (JAK-2) (Figure 1A). The binding of the hormone promotes dimerization of the receptor (Ob-Rb) and phosphorylation in tyrosine of JAK-2 molecules, which, eventually, catalyze phosphorylation of receptors (Ob-Rb) in tyrosine residues, joining and activating (through the catalytic action of JAK-2) proteins of the STAT (signal transducer and transcription activator) family, particularly STAT-3. IL-6, transiently inflammatory,^(7)^ by acting on its homologous receptor (GP130), activates in parallel STATS through the JAK-2 (Figure 1B). From the transcription generated by STAT, cytokine signaling suppression protein or SOCS emerges. SOCS-3 stops the signals initiated by the activation of the leptin receptor.^(7,10)^ TNF-α, through its receptor (TNFR1), activates intracellular substrates that participate in the control of the transcription of inflammatory response genes. One of the main intermediate substrates of the TNF-α signaling pathway is serine kinase JNK (c-Jun N-terminal kinase), that primarily activates the formation of the dimeric transcription factor activator of proteín-1 (AP-1). Mediated by JNK, AP-1 intensifies an inflammatory signaling when transcribing, for example, genes of IL-1β, IL-6 and TNF-α. Moreover, it transcribes enzymes with the capacity to degrade the components of the extracellular matrix of the cartilage (metalloproteinases − MMPs), especially of collagen type II (Figure 1C).^(11)^ TNF-α, IL-6 and IL-1β act through independent pathways that, however, converge to common points, culminating in the activation of nuclear transcription factor Kappa beta (NF-kβ), that transcribes the genes of the inflammatory cytokines themselves − IL-1β, IL-6 and TNF-α, in addition to induced nitric oxide -synthase (iNOS), perpetuating the inflammatory cycle (Figure 1D).^(7,12)^ Another condition responsible for triggering low grade inflammatory response is the exaggerated consumption of saturated fat-rich foods, like milk, meat and cheeses, among other of animal origin. Saturated lauric, myristic and palmitic fatty acids contribute to the development of hypothalamic resistance to leptin and insulin, because they activate receptors of the immune system, integrated to the metabolic system. Toll-like type receptors, TLR4, (Figure 1E) traditionally activated by bacterial lipopolysaccharides (LPS) can also be activated by these fatty acids, simulating the infectious process, triggering a pro-inflammatory response.^(13)^ In this way, low grade inflammation, once installed, is self-limited by the presence of cytokines produced and released by the fatty tissue itself.

During the second stage of the disease, resistance to insulin and to leptin is spread to peripheral systems, perpetuating the hyperglycemic state. The condition predisposes to the development of DM2 and also contributes to the increase in fat mass and continuity of inflammation, changing the cell signal pathways of some organs, such as the liver, skeletal muscles, lungs, endothelium and articular cartilages.^(8)^ Due to the inflammatory capacity acquired by leptin when produced in high amounts, several studies reported it as the initial factor in the genesis of other peripheral inflammatory disorders such as OA.^(14)^

In this case, the genesis of OA would be the inflammatory process initiated at the articular cartilage, as a consequence of the high level of inflammatory components circulating in obese individuals, mainly IL-1β and TNF-α.^(15-20)^ Dumond et al.^(21)^ showed that the level of leptin in the cartilage and synovial fluid of individuals with OA is higher when compared to healthy individuals. In the cartilage, leptin, associated with classic inflammatory cytokines of OA, such as IL-1β and TNF-α, has a catabolic function, increasing the expression of proteolytic enzymes, such as MMPs 1 and 13 and aggrecanases (ADAMTS 4 and 5). The expression of SOCS3 (cytokine 3 signaling suppressor protein of the final signaling pathway of leptin) is increased in the articular cartilage of OA patients, but correlates positively with the expression of other genes, such as MMP-13 and a ADAMTS-5, and is inversely related to the expression of type II collagen. Therefore, SOCS3 (cytokine 3 signaling suppressor) regulates genes involved in cartilage degradation, while it decrease the expression of type II collagen.^(22-24)^

### Relation between obesity and osteoarthritis

**Chart 1:**
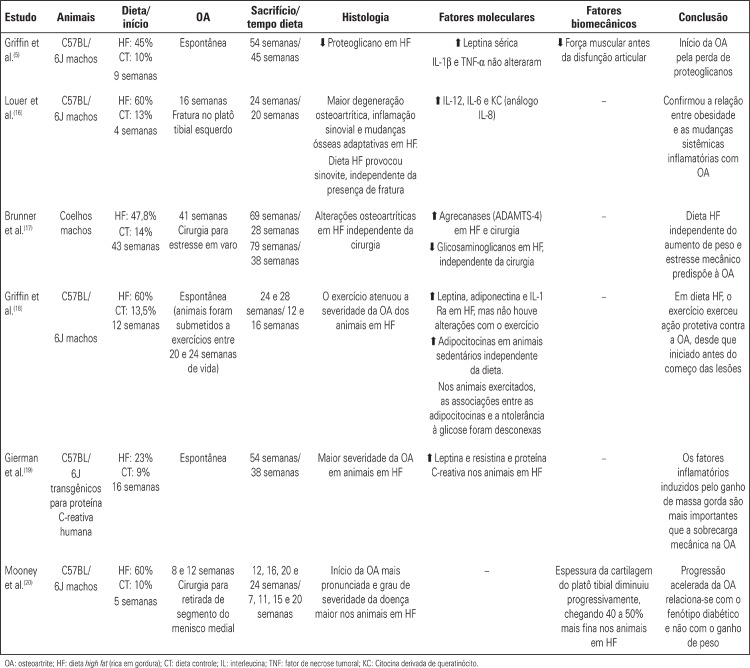
. Main experimental studies with phenotypes of obesity and type 2 *diabetes mellitus*, induced by a saturated fat-rich diet (high lipid content), and their relations with onset and progression of osteoarthritis

In order to attempt to understand the mechanisms involved in the relation between obesity and the pathogenesis of OA, several experimental studies have been proposed, using animals with the phenotype of obesity and DM2 induced by a saturated fat-rich diet. The development and progression of the disease were evaluated according to histological and molecular criteria. The main studies and their results are in chart 1.

Despite the discrepancies among diet compositions (percentage of fraction of saturated fat – 40 to 50%); age at which animals were submitted to the diet (4 or 16 weeks); and also how osteoarthritic alterations were obtained (spontaneous or surgically induced), conclusions are similar. There is a relation between obesity and systemic inflammatory status that, in turn, provide articular chondrocytes with increased expression of MMPs and aggrecanases, with consequent reduction in the expression of collagen and proteoglycans. Thus, there is a homeostatic imbalance of cartilage, favoring the initial osteoarthritic inflammation. And the composition of the fat rich diet, regardless of increase in weight and consequent mechanical overload on joints, predisposes to emergence of OA.^(15-21)^ In common in these studies was the increase in concentration of serum leptin with a saturated fat-rich diet. Increased body adiposity, in itself, cannot be considered a risk factor for the development of OA, given animals with deficient production of this hormone, even on a high lipid diet and increased weight, did not develop disease. This shows the importance of leptin in the inflammatory process of OA, in addition to the well-known inflammatory cytokines.^(15)^

In order to prove the inflammatory action of leptin in OA, in a study with bovine nasal chondrocytes, leptin was associated with IL-1β and TNF-α, increasing the expression of MMPs. Moreover, leptin induces the transcription of SOCS3 that prevents that the anti-inflammatory signals triggered by IL-10 be executed. The homology of leptin for the receptors of the superfamily of cytokines culminates in the activation of the IL-6 receptor, which is capable of transducing its signals to the JNK and IkBkinase (IKK) routes, both beginning and intensifying the activation of NF-kβ, as mentioned previously, responsible for the transcription of genes of inflammatory proteins. Based on these studies, we can suggest that hypertrophic white fat tissue induces MMPs mainly through leptin and, thus, obesity relates directly to triggering OA.^(22)^

### Treatment perspectives: exercise and diet

OA does not have cure perspectives yet, and modern studies using stem-cells for replacing cartilage tissue are, for the time being, distant from being plausible.^(23)^ Treatment has been based on pain control, and on the dysfunction and control of the speed of the cartilage destruction process mainly by taking medication and by exercising. However, prescribing exercise to OA patients is controversial. Some studies showed that exercising may impair the cartilage degradation process due to the increase in mechanical overload,^(24)^ but, on the other hand, evidence has shown that there are no correlations between exercise and OA,^(25)^ or even that exercise is effective for its treatment.^(26)^ Nonetheless, all these studies have observed the changes caused by joint overload. The present revision emphasizes the mechanism of OA genesis directly related to the level of circulating leptin in obesity. This weight excess can be controlled and even reverted upon improvement of eating habits and by including exercise in daily routine. Even on a fat rich diet, exercising promotes protection against OA, given it begins in initial lesions.^(18)^ Hypothalamic sensitivity to leptin also improves with exercising. This is because skeletal muscle releases IL-6 during physical activity and, at the central level, IL-6 regulates energy expenditure, appetite and body composition, decreasing inflammation and increasing sensitivity to leptin.^(27)^

Evidence suggests that the control of the inflammatory parameters of obesity could prevent the emergence of OA or even curb its aggressiveness. Besides exercising, new eating habits aim to change diet composition, given saturated fatty acids are the main factor responsible for low grade inflammation.

Non-saturated fatty acids, mainly omega 3 and omega 9 have been proven to have anti-inflammatory effects in animals by reverting high fat diet-induced hypothalamus inflammation and body adiposity.^(28)^ Evidence of the anti-inflammatory potential of these fatty acids have also been related to OA. In omega 3 treated chondrocyte culture, there was less expression of aggrecanases and cytokines.^(29)^

## CONCLUSION

Obesity is a risk factor for osteoarthritis and the growth in fat mass is directly proportional to exaggerated consumption of nutrients, especially saturated fatty acids, responsible for low grade inflammation and central resistance to insulin and to leptin. At high levels, leptin becomes inflammatory and can trigger an inflammatory process in articular cartilage, changing the homeostasis of this tissue. Exercising and changing diet composition, such as replacing fat by non-saturated fatty acids, can revert the inflammatory process and resistance to leptin, attenuating the speed of progression or preventing the emergence of osteoarthritis.
