# Supplementary material for: Obesity versus osteoarthritis: beyond the mechanical overload
Source: Einstein (Sao Paulo). 2014 Jul-Sep;12(3):374–9. doi: 10.1590/S1679-45082014RB2912 (PMC4872954; doi:10.1590/S1679-45082014RB2912)
Supplement: Translation

# Translation: Obesidade *versus* osteoartrite: muito além da sobrecarga mecânica

**Authors:** Angélica Rossi Sartori-Cintra, Priscila Aikawa, Dennys Esper Correa Cintra

**Affiliations:** 1 Faculdade Anhanguera de Campinas, Campinas, SP, Brasil;; Faculdade Anhanguera de Indaiatuba, Indaiatuba, SP, Brasil.; 2 Faculdade Anhanguera de Rio Grande, Rio Grande, RS, Brasil.; 3 Faculdade de Ciências Aplicadas, Universidade Estadual de Campinas, Limeira, SP, Brasil.

**Keywords:** Osteoartrite, Obesidade/complicações, Inflamação, Leptina, Cartilagem articular

## Abstract

Atualmente, a obesidade é considerada o maior problema de saúde pública do mundo, já atingindo características epidêmicas, segundo a Organização Mundial da Saúde. O acúmulo excessivo de peso é o maior fator de risco, associado a diversas doenças, como *diabetes mellitus* tipo 2, hipertensão, dislipidemias e doenças osteometabólicas, como osteoporose e osteoartrite. A osteoartrite é a doença reumática mais prevalente, e a principal causa de incapacidade física e diminuição da qualidade de vida da população acima de 65 anos. Acomete principalmente as articulações que suportam peso, como joelhos e quadris. No entanto, juntamente com os casos de obesidade, sua prevalência vem aumentando e em outras articulações, como as das mãos. Assim, supõe-se que a influência da obesidade no desenvolvimento da osteoartrite esteja além da sobrecarga mecânica. O objetivo desta revisão foi correlacionar os possíveis mecanismos que determinam a gênese e o desenvolvimento dessas duas doenças. O aumento da massa adiposa é diretamente proporcional ao consumo exagerado de ácidos graxos saturados, responsáveis pela condição sistêmica de inflamação de baixo grau e resistência à insulina e à leptina. Em níveis elevados, a leptina assume características inflamatórias e age na cartilagem articular, desencadeando o processo inflamatório e alterando a homeostase desse tecido com consequente degeneração. Conclui-se que a obesidade é um fator de risco para a osteoartrite e que a prática de atividade física e modificações na composição da dieta podem reverter o quadro inflamatório e a resistência à leptina, atenuando a progressão ou prevenindo o surgimento da osteoartrite.

## INTRODUÇÃO

Segundo a Organização Mundial da Saúde (OMS), a obesidade já assume características epidêmicas, atingindo mais de 1 bilhão de adultos no mundo. Trata-se do maior fator de risco associado a diversas doenças, como resistência à insulina e *diabetes mellitus* tipo 2 (DM2), hipertensão, dislipidemias e alguns tipos de câncer. Secundariamente, a obesidade está fortemente associada a uma série de distúrbios, como asma e esteato-hepatite não alcoólica, além de doenças osteometabólicas, como a osteoporose e a osteoartrite.^(1)^ A osteoartrite (OA) é a doença reumática osteometabólica, mais prevalente, constituindo a principal causa de incapacidade física e diminuição da qualidade de vida da população acima de 65 anos, sendo caracterizada pela degradação da cartilagem articular.^(2)^

Historicamente, as relações feitas entre a obesidade e o desenvolvimento da OA restringiam-se às alterações biomecânicas da articulação, provocadas pelo aumento do peso corporal, levando à gênese de um processo inflamatório na cartilagem, e culminando no desenvolvimento e progressão da doença. Essas alterações ocorrem preferencialmente em articulações que suportam o peso corporal, como joelho e quadris.^(3)^ No entanto, juntamente do aumento da prevalência da obesidade na população mundial, houve também aumento dos casos de OA em articulações que não são responsáveis por suporte de peso, como as articulações das mãos e temporomandibulares (ATMs).^(4)^ Dessa maneira, supõe-se que a influência da obesidade no desenvolvimento da OA esteja além da sobrecarga na articulação pelo aumento do índice de massa corporal (IMC).^(5)^

Assim, diversos estudos têm sido desenvolvidos para desvendar os mecanismos envolvidos na relação entre obesidade e OA. O objetivo desta revisão foi agrupar as evidências mais recentes sobre os possíveis mecanismos moleculares, correlacionando as duas doenças.

### Fisiopatologia da obesidade e da osteoartrite

Na obesidade, a hipertrofia do tecido adiposo aumenta a expressão e a liberação das chamadas adipocitocinas, como a interleucina (IL) 1β, IL-6, resistina, leptina e fator de necrose tumoral alfa (TNF-α), que são necessárias para a diferenciação celular e hematopoiese, entre outras funções. Contudo, tais funções dependem de sua concentração na circulação sanguínea. A elevação para um grau “superótimo” já é capaz de dar início ao processo inflamatório de baixo grau.^(6,7)^

Essas proteínas atingem a corrente sanguínea e alcançam o hipotálamo, no sistema nervoso central, interferindo na fina regulação exercida por ele, ao controlar a fome e o gasto energético. Esse desajuste o torna resistente aos hormônios periféricos insulina e leptina, principalmente.^(8)^ Dessa maneira, o indivíduo apresenta descontrole da saciedade, aumentando a ingestão de alimentos, com consequente aumento do peso e do tecido adiposo, que, por sua vez, libera ainda mais leptina, atingindo o ponto de hiperleptinemia. Em concentrações elevadas, a leptina assume característica de cunho inflamatório. Sua estrutura apresenta homologia aos receptores das citocinas inflamatórias (IL-1 e IL-6), sendo considerada, portanto, como integrante da superfamília de citocinas.^(7,9)^

A leptina se liga ao seu receptor (Ob-Rb), que se encontra constitutivamente associado à molécula Janus Kinase-2 (JAK-2) (Figura 1A). A união do hormônio promove dimerização do receptor (Ob-Rb) e a fosforilação em tirosina das moléculas de JAK-2, que, finalmente, catalisam a fosforilação dos receptores (Ob-Rb) em resíduos tirosina, reunindo e ativando (por meio da ação catalítica da JAK-2) proteínas da família STAT (transdutor de sinal e ativador da transcrição), particularmente a STAT-3. A IL-6, transitoriamente inflamatória,^(7)^ agindo sobre seu receptor homólogo (GP130), ativa paralelamente as STATS pela JAK-2 (Figura 1B). Da transcrição gerada pela STAT, surge a proteína de supressão da sinalização de citocinas ou SOCS. A SOCS-3 encerra os sinais iniciados pela ativação do receptor de leptina.^(7,10)^ O TNF-α, por meio de seu receptor (TNFR1), ativa substratos intracelulares que participam do controle da transcrição de genes de reposta inflamatória. Um dos principais substratos intermediários da via de sinalização do TNF-α é a serinaquinase JNK (c-Jun N-terminal Kinase), a qual primariamente ativa, no núcleo celular, a formação do fator de transcrição dimérico ativador de proteína-1 (AP-1). AP-1, mediada por JNK, intensifica a sinalização inflamatória ao transcrever, por exemplo, genes da IL-1β, IL-6 e TNF-α. Além disso, transcreve também enzimas com capacidade de degradação dos constituintes da matriz extracelular da cartilagem (metaloproteinases − MMPs), especialmente do colágeno tipo II (Figura 1C).^(11)^ TNF-α, IL-6 e IL-1β agem por meio de vias independentes que, no entanto, convergem para pontos em comum, culminando na ativação do fator de transcrição nuclear Kappa beta (NF-kβ), que transcreve os genes das próprias citocinas inflamatórias − IL-1β, IL-6 e TNF-α, além da óxido nítrico-sintase induzida (iNOS), perpetuando o ciclo inflamatório (Figura 1D).^(7,12)^ Outra condição responsável pelo desencadeamento da resposta inflamatória de baixo grau é o consumo exagerado de alimentos ricos em gorduras saturadas, como leite, carne e queijos, entre outros de origem animal. Os ácidos graxos saturados láurico, mirístico e palmítico contribuem para a evolução da resistência hipotalâmica à leptina e insulina, por ativarem receptores de sistema imune, integrados com o sistema metabólico. Os receptores do tipo *Toll-like* 4 (*toll-like receptors*, TLR4) (Figura 1E) tradicionalmente ativados por lipopolissacárides (LPS) bacterianos podem ser ativados também por esses ácidos graxos, mimetizando o processo infeccioso, disparando resposta pró-inflamatória.^(13)^ Dessa maneira, inflamação de baixo grau, uma vez instalada, é autoalimentada pela presença das citocinas produzidas e liberadas pelo próprio tecido adiposo.

**Figura 1:** A leptina, por meio de seu receptor ObR (A) e as vias da interleucina 6, por meio do seu receptor GP-130 (B), ativam o fator de transcrição STAT3 que, no núcleo, transcreve o gene da SOCS3, com função de suprimir as vias de sinalização da leptina. No entanto, ativa também a transcrição de metaloproteinases e agrecanases, proteínas de degradação da cartilagem. Vias de sinalização das citocinas inflamatórias: fator de necrose tumoral alfa por meio do receptor de fator de necrose tumoral (C), interleucina 1β por seu receptor interleucina 1β (D), e dos ácidos graxos saturados, que são reconhecidos pelos receptores *Toll-like* 4 (E). Essas vias culminam na ativação dos fatores de transcrição NF-kβ e AP-1, que transcrevem os genes de proteínas inflamatórias (interleucina 1β, interleucina 6, fator de necrose tumoral alfa, óxido nítrico-sintase induzida, entre outros)

Numa segunda etapa da doença, a resistência à insulina e à leptina é propagada aos sistemas periféricos, perpetuando o estado hiperglicêmico. Essa condição predispõe ao desenvolvimento do DM2 e também contribui para o aumento da massa adiposa e manutenção do estado inflamatório, alterando as vias de sinalizações celulares de órgãos como fígado, musculatura esquelética, pulmões, endotélio e cartilagens articulares.^(8)^ Pela capacidade inflamatória adquirida pela leptina quando produzida em altas quantidades, diversos estudos relatam este como o fator inicial da gênese de outros distúrbios inflamatórios periféricos, como, por exemplo, a OA.^(14)^

Nesse caso, a gênese da OA estaria no processo inflamatório iniciado na cartilagem articular, como consequência do nível elevado dos componentes inflamatórios circulantes em indivíduos obesos, principalmente IL-1β e TNF-α.^(15-20)^ Dumond et al.^(21)^ demonstraram que o nível de leptina na cartilagem e líquido sinovial de indivíduos com OA é maior quando comparado ao de indivíduos saudáveis. Na cartilagem, a leptina, associada às citocinas inflamatórias clássicas da OA, como IL-1β e TNF-α, tem função catabólica, aumentando a expressão de enzimas proteolíticas, como as MMPs 1 e 13 e as agrecanases (ADAMTS 4 e 5). Em pacientes com OA, a expressão da SOCS3 (supressor de sinalização de citocinas 3, proteína da via final de sinalização da leptina) está aumentada na cartilagem articular, mas se correlaciona positivamente com a expressão de outros genes, como a MMP-13 e a ADAMTS-5, e é inversamente relacionada com a expressão do colágeno tipo II. Portanto, a SOCS3 (supressor de sinalização de citocinas 3) regula genes envolvidos na degradação da cartilagem, enquanto diminui a expressão de colágeno tipo II.^(22-24)^

### Relação entre obesidade e osteoartrite

Para tentar compreender os mecanismos envolvidos nessa relação entre obesidade e patogênese da OA, diversos estudos experimentais têm sido propostos, utilizando animais com fenótipo de obesidade e DM2 induzidos por dieta rica em gordura saturada – hiperlipídica. O desenvolvimento e a progressão da doença foram avaliados segundo critérios histológicos e moleculares. Os principais estudos e seus resultados estão no quadro 1.

**Quadro 1:** Principais estudos experimentais com fenótipo de obesidade e *diabetes mellitus* tipo 2, induzidos por dieta rica em gordura saturada (hiperlipídica), e suas relações com o surgimento e a progressão da osteoartrite

Apesar das discrepâncias entre as composições das dietas (percentagem da fração de gordura saturada – 40 a 50%); idade em que os animais foram submetidos a essa dieta (4 e 16 semanas); e também da maneira como foram obtidas as alterações osteoartríticas (espontânea ou cirurgicamente induzidas), as conclusões são similares. Há relação entre obesidade e o *status* inflamatório sistêmico, que, por sua vez, proporciona aos condrócitos articulares aumento na expressão das MMPs e agrecanases, com consequente redução na expressão de colágeno e proteoglicanos. Dessa maneira, ocorre desequilíbrio homeostático da cartilagem, favorecendo o início da inflamação osteoartrítica. Assim, a composição da dieta, rica em gordura, independentemente do aumento de peso e consequente sobrecarga mecânica nas articulações, predispõe ao surgimento da OA.^(15-21)^ Também foi comum, nesses estudos, o aumento das concentrações da leptina sérica com dieta rica em gordura saturada. O aumento da adiposidade corporal, por si só, não pode ser considerado fator de risco para o desenvolvimento da OA, uma vez que animais com produção deficiente desse hormônio, mesmo sob dieta hiperlipídica e aumento de peso, não desenvolveram a doença. Isso caracteriza a importância da leptina no processo inflamatório da OA, além das conhecidas citocinas inflamatórias.^(15)^

Para comprovar a ação inflamatória da leptina na OA, num estudo com condrócitos nasais bovinos, a leptina associou-se a IL-1β e TNF-α, aumentando a expressão das MMPs. Além disso, a leptina induz à transcrição da SOCS3, que impede que os sinais anti-inflamatórios disparados pela IL-10 sejam executados. A homologia da leptina pelos receptores da superfamília das citocinas culmina na ativação do receptor da IL-6, que é capaz de transduzir seus sinais para as vias da JNK e IkBkinase (IKK), ambas iniciando ou intensificando a ativação do NF-kβ, como dito anteriormente, responsáveis pela transcrição de genes de proteínas inflamatórias. Por esses estudos, podemos sugerir que o tecido adiposo branco (TAB) hipertrófico induz às MMPs por meio, principalmente da leptina e, dessa forma, a obesidade relaciona-se diretamente com o desencadeamento da OA.^(22)^

### Perspectivas de tratamentos: exercícios e dieta

A OA ainda não apresenta perspectivas de cura, e os modernos estudos utilizando células-tronco para reposição do tecido cartilaginoso encontram-se, por enquanto, distantes de serem plausíveis.^(23)^ O tratamento tem se baseado no controle da dor, na disfunção e no controle da velocidade do processo de destruição da cartilagem, feito principalmente pela administração de medicamentos e pela prática de exercícios. No entanto, a prescrição de exercícios aos pacientes com OA é controversa. Estudos demonstram que a prática de exercícios pode prejudicar o processo de degradação da cartilagem em razão do aumento da sobrecarga mecânica,^(24)^ mas, por outro lado, evidências mostram que não existem correlações entre exercícios e OA,^(25)^ ou mesmo que o exercício seja efetivo em seu tratamento.^(26)^ De qualquer forma, todos esses estudos verificaram as alterações provocadas pela sobrecarga articular. Nesta revisão, explicitamos o mecanismo da gênese da OA diretamente relacionado ao nível de leptina circulante na obesidade. Esse excesso de peso pode ser controlado e até revertido com a melhoria de hábitos alimentares e com a inclusão da prática de atividade física na rotina diária. Mesmo sob dieta rica em gordura, o exercício promove ação protetora contra a OA, desde que iniciado antes do início das lesões.^(18)^ A sensibilidade hipotalâmica à leptina também apresentou melhora com a prática de exercícios. Isso porque o músculo esquelético libera IL-6 durante atividade física e, em nível central, a IL-6 regula o gasto energético, o apetite e composição corporal, diminuindo a inflamação e aumentando a sensibilidade à leptina.^(27)^

Essas evidências sugerem que o controle dos parâmetros inflamatórios da obesidade poderia prevenir o surgimento da OA ou mesmo diminuir sua agressividade. Além dos exercícios, os novos hábitos alimentares visam à modificação da composição da dieta, uma vez que os ácidos graxos saturados são os principais responsáveis pela inflamação de baixo grau.

Ácidos graxos insaturados, principalmente os ômega 3 e ômega 9 demonstraram, em animais, efeitos anti-inflamatórios, ao reverterem a adiposidade corporal e a inflamação hipotalâmica induzida por dieta hiperlipídica.^(28)^ Evidências do potencial anti-inflamatório desses ácidos graxos insaturados também foram relacionados com a OA. Em cultura de condrócitos tratados com ômega 3, houve menor expressão das agrecanases e citocinas.^(29)^

## CONCLUSÃO

A obesidade é um fator de risco para a osteoartrite e o aumento da massa adiposa é diretamente proporcional ao consumo exagerado de nutrientes, especialmente os ácidos graxos saturados, responsáveis pela condição de inflamação de baixo grau e resistência central à insulina e à leptina. Em níveis elevados, a leptina assume características inflamatórias e pode desencadear um processo inflamatório na cartilagem articular, alterando a homeostase desse tecido. A prática de atividade física e as modificações na composição da dieta, como substituição da gordura por ácidos graxos insaturados, podem reverter o quadro inflamatório e a resistência à leptina, atenuando a velocidade de progressão ou prevenindo o surgimento da osteoartrite.
